# Predictors of Normalization of Circulating Interleukin-6 after Cardiac Myxoma Resection

**DOI:** 10.21470/1678-9741-2018-0161

**Published:** 2019

**Authors:** Shi-Min Yuan, Hui-Zhen Lin

**Affiliations:** 1 Department of Cardiothoracic Surgery, The First Hospital of Putian, Teaching Hospital, Fujian Medical University, Putian, Fujian Province, People's Republic of China.; 2 Department of Clinical Investigation, The First Hospital of Putian, Teaching Hospital, Fujian Medical University, Putian, Fujian Province, People's Republic of China.

**Keywords:** Cardiac Surgical Procedures, Interleukin-6, Neoplasms, Myxoma, Heart Neoplasms

## Abstract

**Objective:**

To disclose the relationships between the anatomic features of cardiac myxomas and plasma interleukin (IL)-6 levels.

**Methods:**

Twelve patients undergoing cardiac myxoma resection at The First Hospital of Putian, Teaching Hospital, Fujian Medical University were enrolled into this study. Pre- and postoperative IL-6 levels were determined by an enzyme-linked immunosorbent assay method, and correlations between cardiac myxoma dimension or volume and plasma IL-6 levels were analyzed. C-reactive protein (CRP) levels were also evaluated.

**Results:**

IL-6 and CRP levels were significantly decreased one month after cardiac myxoma resection in comparison to preoperative values. IL-6 and CRP levels did not differ between patients with a cardiac myxoma of irregular appearance and those with a myxoma of regular gross appearance, or between patients with a pedicled or a sessile myxoma. Decrement of IL-6 of patients with irregular cardiac myxomas was much higher than that of patients with regular ones, while no intergroup difference was noted in decrement of CRP. A close direct correlation was noted between IL-6 levels and maximal dimension (length) or volume of cardiac myxomas, whereas CRP levels only correlated with maximal dimension of cardiac myxomas.

**Conclusion:**

Anatomic features of cardiac myxomas (sessile, irregular appearance, maximal dimension, and volume) could be determinants of the patients' circulating IL-6 levels. IL-6 was likely to be a more sensitive biomarker than CRP in predicting the inflammatory status of patients with cardiac myxoma. Sessile and irregular cardiac myxomas might predict more severe inflammatory conditions for their more abundant endothelial cells and IL-6 overproduction.

**Table:** 

Abbreviations, acronyms & symbols	
CRP	= C-reactive protein
IL	= Interleukin
mRNA	= Messenger ribonucleic acid

## INTRODUCTION

Interleukins (ILs) are a group of cytokines involved in the etiologies of many inflammation-related disorders, trigger inflammation-induced immune responses, such as pain, various infections and allergic reactions, and cell regeneration^[1,2]^. Meanwhile, an antibody directed against an IL-specific peptide shows anti-inflammatory functions^[3]^. Of the IL family, IL-6 is a pro- and anti-inflammatory cytokine, responsible for the pathogenesis of many inflammation-related conditions, including infectious^[1,4-6]^ and immunological disorders^[2,7]^. A recent review comprehensively described the profile of circulating IL-6 in relation to different cardiac operations^[8]^. As a response to the acute phase of an inflammation, IL-6 has been proved to be an indicator of immunologic abnormalities and constitutional symptoms of cardiac myxoma^[9]^. Clinical observations revealed that circulating IL-6 levels closely correlated with the dimensions of cardiac myxomas^[9]^, and that IL-6 levels could be reduced or diminished shortly after surgical resection of a cardiac myxoma^[9]^. Soeparwata et al.^[10]^ and Mendoza et al.^[9]^ found a positive correlation between the cardiac myxoma size index (*i.e*., tumor volume) and circulating IL-6 levels. We assumed that, besides the relationship between tumor size (maximal dimension [length] and volume) and IL-6, there might also be close relations between IL-6 or decrement (difference of pre- and postoperative values) of IL-6 (∆IL-6) and the anatomic features of cardiac myxomas. In order to highlight these hypotheses, we made a retrospective study on a small patient population undergoing cardiac myxoma resection to evaluate circulating IL-6 levels in parallel with C-reactive protein (CRP) levels.

## METHODS

In total, 12 patients undergoing cardiac myxoma resection at The First Hospital of Putian, Teaching Hospital, Fujian Medical University were enrolled into this study. There were 9 (75%) female and 3 (25%) male patients. Their ages were 53.3 ± 6.1 (range, 46-66; median, 51) years. No age difference was found between genders (51.4 ± 3.7 *vs.* 58.7 ± 9.5, *P*=0.0723).

All 12 patients were diagnosed with left atrial myxoma by transthoracic echocardiography and it was verified by surgical operation and pathological examinations of the resected tumors. They underwent cardiac myxoma resection with the aid of cardiopulmonary bypass via a left atriotomy or a right atriotomy plus an atrial septotomy approach. All patients survived the operation and were followed up. No recurrence of cardiac myxoma was found in any patient during the follow-up period.

Of the 12 patients, 11 (91.7%) had 1 cardiac myxoma and 1 (8.3%) had 1 big and 2 very small myxomas, totaling 14 myxomas in all of them. Thirteen left atrial myxomas in 11 (91.7%) patients were arising from the atrial septum (5 of them from the fossa ovalis) and 1 myxoma in 1 (8.3%) patient was arising from the free wall of the left atrium. Seven (50%) myxomas were pedicled and 7 (50%) were sessile. Except for the 2 very small myxomas, the gross appearances of the remaining 12 myxomas were regular in 7 (58.3%) patients and irregular in 5 (41.7%). One of the regular myxomas was capsulated with dense membrane, and 1 of the irregular myxomas was villious. Patients' demographics were listed in Table 1.

**Table 1:** Patients' demographics.

Variable		Result
Age, year		53.3±6.1 (range, 47-66; median, 50.5)
Gender, male/female		3/9
Major onset manifestation		Dyspnea (n=5), fatigue (n=3), fever (n=1), hiccups (n=1), vertigo and paresthesia of the extremities (n=1), acute cerebral infarct (n=1)
Tumor pathology	Tumor number	14
	Singularity/multiplicity	Solitude (n=11), multiple (n=1)
	Gross appearance	Regular (n=7), irregular (n=5)
	Attachment	Pedicled (n=7), sessile (n=7) (1 [8.3%] patient had 1 big and 2 very small sessile myxomas)
Tumor dimensions, mm	Length	50.0±12.9 (range, 35-76; median, 47.5)
	Width	36.1±8.4 (range, 25-46; median, 38.5)
	Height	25.2±10.2 (range, 10-38; median, 27.5)
	Tumor volume, mm^3^	53722.1±34940.7 (range, 10750-114380; median, 59515)
IL-6, pg/mL	Preoperative	15.7±6.4 (range, 7.7-28; median, 15.1)
	One-month postoperative	2.2±1.9 (range, 0-5.2; median, 1.3)
	∆IL-6	13.5±6.9 (range, 4.2-27.7; median, 14.1)
CRP, mg/L	Preoperative	49.6±30.2 (range, 17.8-120.9; median, 37.8)
	One-month postoperative	7.1±1.8 (range, 3.4-9.4; median, 7.5)
	∆CRP	42.4±29.3 (range, 12.9-110.6; median, 31.9)

∆CRP=decrement of C-reactive protein; ∆IL-6=decrement of interleukin-6; CRP=C-reactive protein; IL-6=interleukin-6

Blood samples were taken from each patient before operation and at 1-month follow-up after the operation. Pre- and postoperative IL-6 levels were determined by an enzyme-linked immunosorbent assay method. CRP levels were also detected and evaluated.

All data were expressed in mean ± standard deviation, and intergroup comparisons were made by Student *t*-test. Correlations between tumor dimensions and IL-6 levels were assessed as well. *P*<0.05 was considered of statistical significance.

This study was approved by the institutional Ethical Committee and it was conducted following the guidelines of the Declaration of Helsinki. Informed consent was obtained from each patient.

## RESULTS

### IL-6 and CRP Levels

**Fig. 1:**
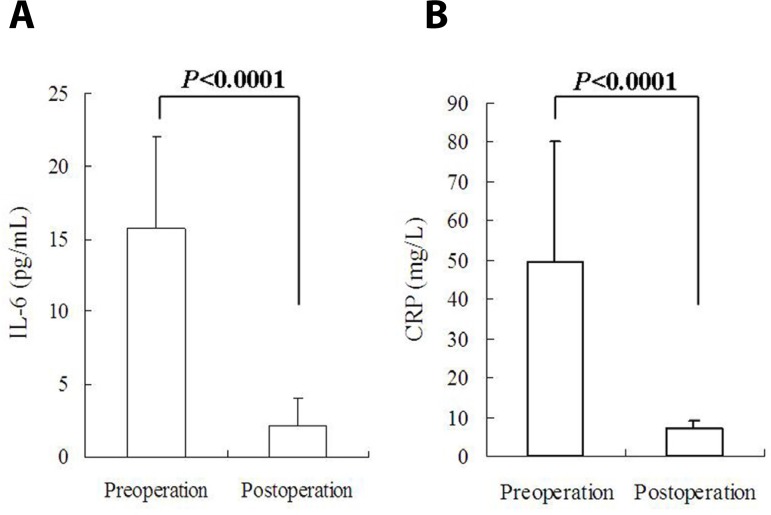
Circulating inflammatory biomarkers were significantly lower after cardiac myxoma resection than preoperative baseline: (A) interleukin-6 (IL-6); and (B) C-reactive protein (CRP).

Preoperative plasma IL-6 level was much higher than the reference value, and IL-6 levels 1 month after cardiac myxoma resection were all in normal range. Postoperative IL-6 level was significantly lower than preoperative value (15.7±6.4 pg/mL *vs.* 2.2±1.9 pg/mL, *P*<0.0001; Figure 1A). Preoperative IL-6 levels between patients without and with constitutional symptoms did not differ statistically (15.6±8.2 pg/mL *vs*. 15.8±5.6 pg/mL, *P*=0.9548).

Preoperative CRP levels were elevated in all patients with a mean of 49.6±30.4 (range, 17.8-120.94; median, 37.13) mg/L. Postoperative CRP levels were significantly decreased in comparison to the preoperative values (Figure 1B). Postoperative CRP value was above the reference value in 3 (25%) patients and normal in 9 (75%).

**Fig. 2:**
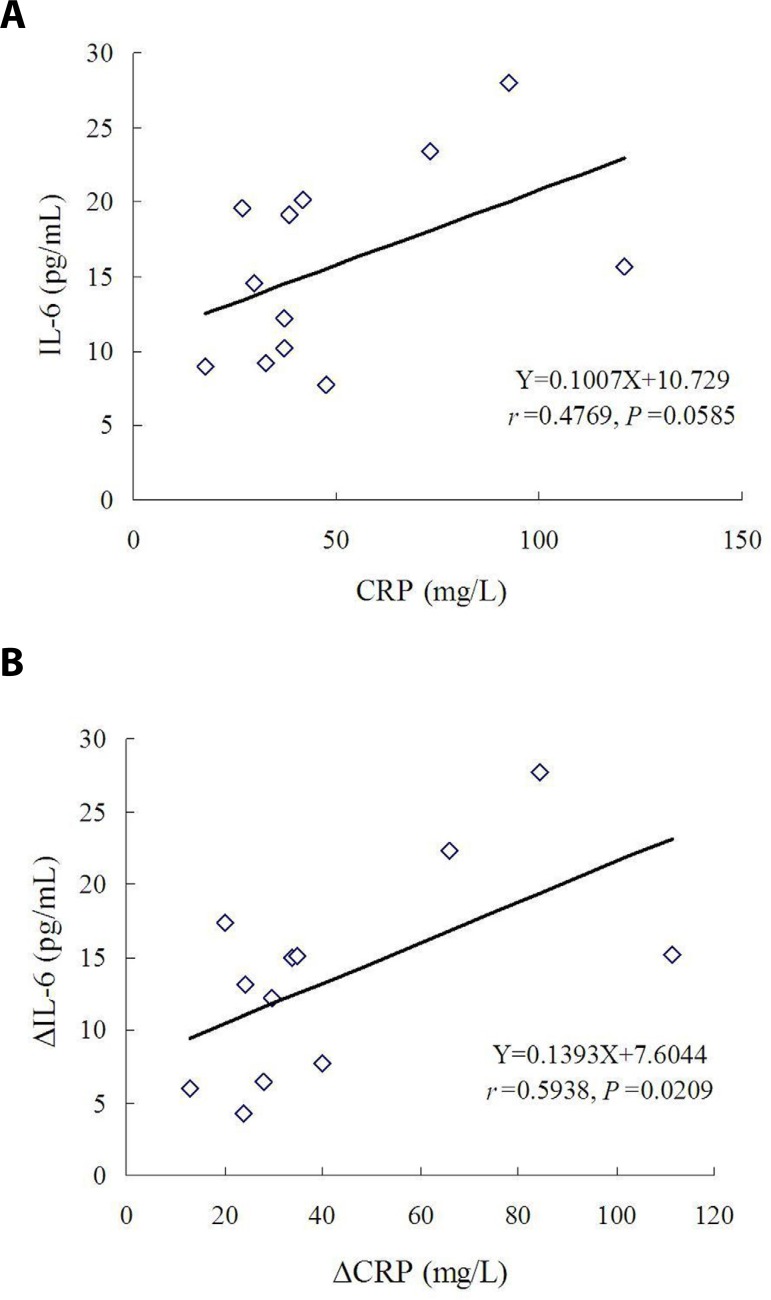
Correlation between circulating interleukin-6 (IL-6) and C-reactive protein (CRP): (A) there was an approximate direct correlation between preoperative IL-6 and preoperative CRP; and (B) there was a close correlation between decrement of interleukin-6 (ΔIL-6) and decrement of C-reactive protein (ΔCRP) values.

There was an approximate direct correlation between preoperative IL-6 and preoperative CRP (r=0.4769, *P*=0.0585; Figure 2A). No correlation was found between postoperative IL-6 and postoperative CRP (r=0.0173, *P*=0.4787). Nevertheless, there was a close correlation between ∆IL-6 and decrement of CRP (∆CRP) (r=0.5938, *P*=0.0209; Figure 2B).

### Tumor Size

**Fig. 3:**
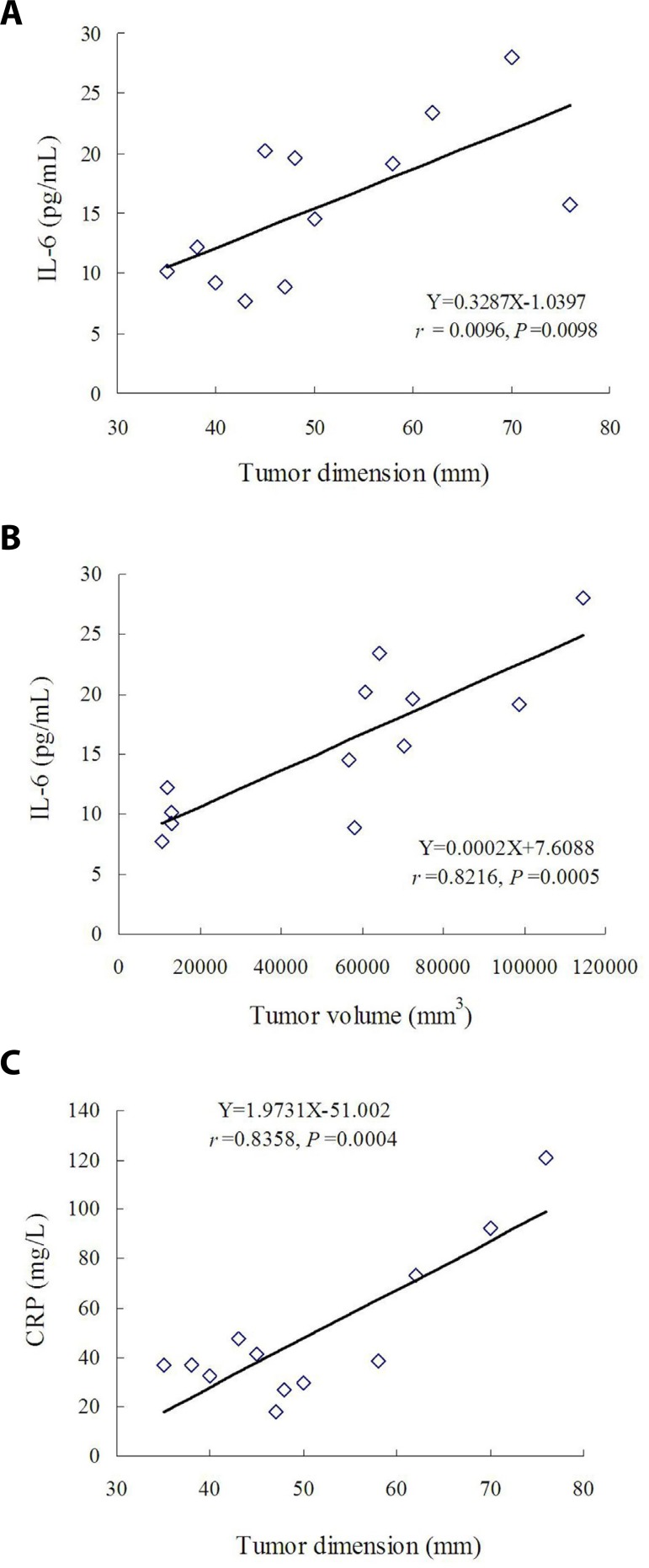
Correlation between preoperative circulating biomarkers and tumor size: (A) close direct correlation between preoperative interleukin-6 (IL-6) levels and maximal dimension of cardiac myxoma; (B) close direct correlation between preoperative IL-6 levels and volume of cardiac myxoma; and (C) close direct correlation between preoperative C-reactive protein (CRP) levels and maximal dimension of cardiac myxoma.

A close direct correlation was noted between preoperative IL-6 levels and the maximal dimension of cardiac myxoma (Figure 3A) or tumor volume (Figure 3B). No significant correlationships were found between ∆IL-6 and maximal dimension of tumor (r=0.0424, *P*=0.4480) or tumor volume (r=0.0574, *P*=0.4297). A close direct correlation was also noted between preoperative CRP levels and maximal dimension of cardiac myxoma (Figure 3C), but no correlation was found between preoperative CRP and tumor volume (r=0.3975, *P*=1.0003).

### Irregular *versus* Regular

**Fig. 4:**
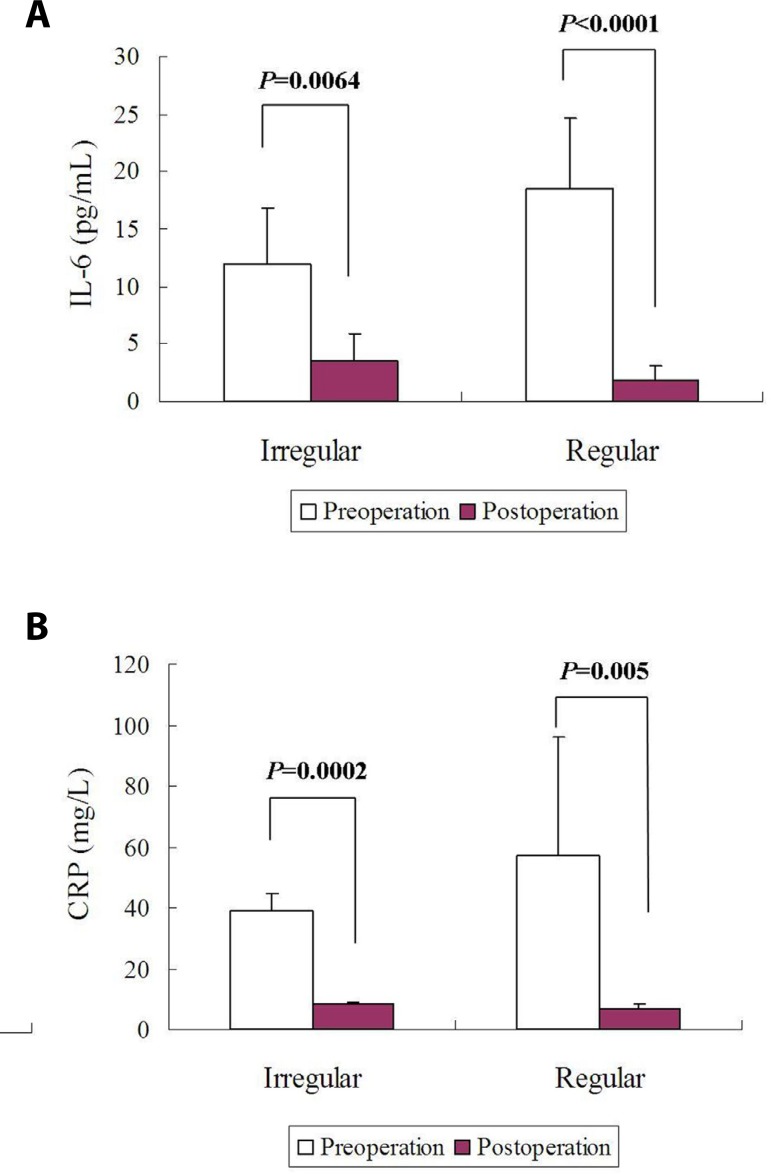
Comparisons of circulating biomarkers between patients with cardiac myxoma of an irregular gross appearance and those with cardiac myxoma of a regular gross appearance: (A) no differences were found in pre- or postoperative interleukin-6 (IL-6) levels between these patients; and (B) no differences were found in pre- or postoperative C-reactive protein (CRP) levels between these patients.

**Fig. 5:**
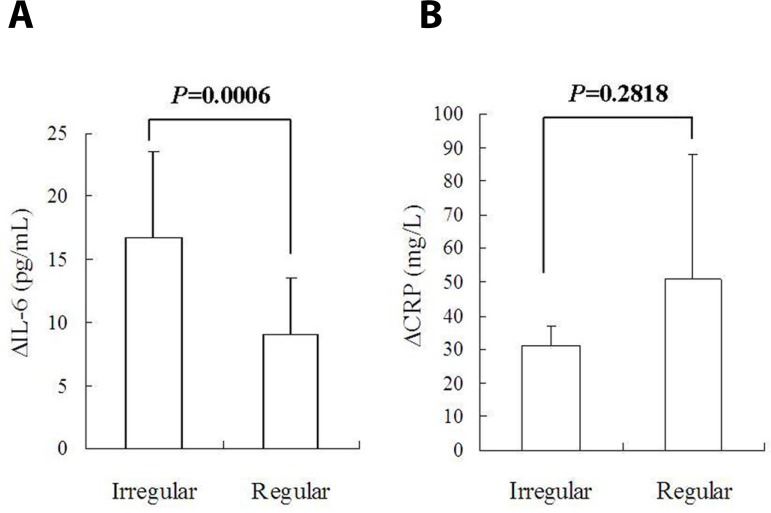
Comparisons of decrements of interleukin-6 (ΔIL-6) and C-reactive protein (ΔCRP) levels: (A) ΔIL-6 of patients with cardiac myxoma of an irregular gross appearance was much higher than that of patients with cardiac myxoma of a regular gross appearance; and (B) no intergroup difference was found in ΔCRP.

Postoperative IL-6 levels were significantly decreased in comparison to the preoperative levels in patients with a cardiac myxoma of either an irregular or a regular gross appearance. No differences were found in pre- or postoperative IL-6 levels of patients with a cardiac myxoma of an irregular gross appearance and those with a regular gross appearance (Figure 4A). The CRP profiles displayed a similar trend to IL-6 (Figure 4B). ∆IL-6 of patients with cardiac myxoma of an irregular gross appearance was much higher than that of patients with cardiac myxoma of a regular one (16.7±6.9 pg/mL *vs*. 9.1±4.4 pg/mL, *P*=0.0454; Figure 5A). No intergroup difference was noted in ∆CRP (Figure 5B).

### Pedicled *versus* Sessile

**Fig. 6:**
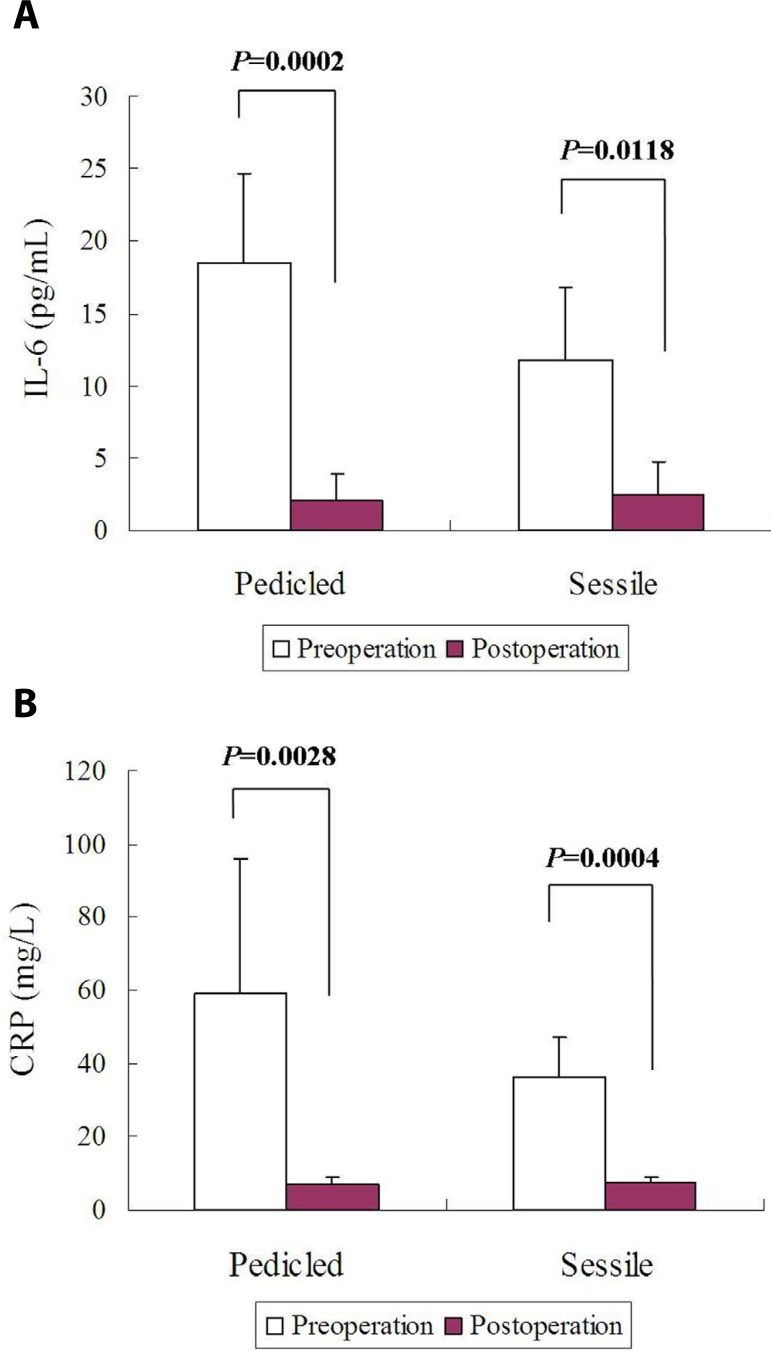
Comparisons of circulating biomarkers between patients with a pedicled or sessile cardiac myxoma: (A) no differences were found in pre- or postoperative interleukin-6 (IL-6) levels between these patients; and (B) no differences were found in pre- or postoperative C-reactive protein (CRP) levels between these patients.

Postoperative IL-6 levels of patients with a pedicled or sessile cardiac myxoma were significantly decreased in comparison to the preoperative levels. No differences were found in pre- or postoperative IL-6 levels of patients with a pedicled and those with a sessile cardiac myxoma (Figure 6A). CRP profiles also displayed a similar trend to IL-6 (Figure 6B). Both ∆IL-6 (16.4±7.4 pg/mL *vs*. 9.5±4.0 pg/mL, *P*=0.064) and ∆CRP (52.2±35.7 mg/L *vs*. 28.9±10.1 mg/L, *P*=0.1912) showed higher results in patients with pedicled cardiac myxoma than in those with sessile myxoma, but there was lack of intergroup differences.

## DISCUSSION

Patients with cardiac myxoma have been reported to have elevated circulating IL-6 levels as a result of the associated inflammatory and immune conditions. Jenni et al.^[11]^ reported that IL-6 levels were elevated in 79% of 19 cardiac myxoma patients. Mendoza et al.^[9]^ reported that the whole group of their 8 patients showed preoperative elevated serum IL-6 levels, and cardiac myxoma resection led to a remarkable reduction of IL-6 in these patients. Clinical observations revealed that not only circulating IL-6, but also IL-6 antigen and IL-6 messenger ribonucleic acid (mRNA), were significantly upregulated in cardiac myxoma tissues as a result of activated immune reactions^[12]^. Seino et al.^[13]^ observed increased expression of IL-6 mRNA in cardiac myxoma tissues of all their 3 patients, and their results supported that IL-6 is overproduced by cardiac myxoma cells. Yaguchi et al.^[14]^ found that both serum and cerebrospinal fluid IL-6 levels were increased in cardiac myxoma patients in whom cerebral aneurysm has been developed. This phenomenon was also observed by other authors^[15,16]^. Elevated IL-6 levels were also associated with occurrence of pertinent sequelae, such as renal injury, proteinuria^[17]^, and constitutional signs^[12]^. Re-elevation of circulating IL-6 was seen in patients with recurrent tumors, and the IL-6 level was even much higher than in the first time occurrence^[10]^. This was explained as more severe cases of cardiac myxoma were usually associated with constitutional symptoms and embolic events^[18,19]^.

The present patient cohort included 12 cases of cardiac myxoma patients; all of them had an elevated plasma IL-6 level preoperatively and a remarkable decrease of IL-6 after cardiac myxoma resection. Only 1 patient developed acute cerebral infarction as a severe complication of cardiac myxoma, while the remaining 11 patients had constitutional or mild neurological symptoms. Although much attention has been drawn that IL-6 plays an important role in producing constitutional symptoms and severe sequalae in patients with cardiac myxoma^[20]^, we did not find any difference in IL-6 levels between patients with and without constitutional symptoms. In addition, we did not find differences in pre- and postoperative IL-6 levels between patients with cardiac myxoma of irregular and regular appearances, neither between patients with a pedicled or a sessile cardiac myxoma. The positive findings of the present study were direct relationships between circulating IL-6 levels and tumor volume or tumor dimension, which were in accordance to the results reported previously by Mendoza et al.^[9]^ and Soeparwata et al.^[10]^. Irregular cardiac myxomas have been proved to have gelatinous and fragile extensions with tendency of spontaneous fragmentation^[21]^ and to be associated with increased risks of myxoma-related embolic events^[22]^. Moreover, regular cardiac myxomas are prone to develop cerebral aneurysms and highly elevated serum IL-6^[16]^. The higher IL-6 levels in patients with irregular myxoma might hint an enlarged tumor surface in charge of more abundant production of cytokines. The higher IL-6 and CRP levels in patients with sessile cardiac myxoma might implicate that more cells or exposed endothelium might remain in the large sessile myxomas.

It has been noted that most patients with cardiac myxoma had leukocytosis or high CRP levels prior to cardiac myxoma resection^[23]^. Only recently, Durgut et al.^[24]^ described in a retrospective study that cardiac myxoma resection led to a significant decrease of CRP from preoperative 47.3±14.6 mg/L to postoperative 8.4±2.6 mg/L period. The inherent relations between IL-6 and CRP in cardiac myxoma patients have been described as that IL-6 could trigger the release of CRP. In line with this theory, we observed close direct relations between preoperative IL-6 and preoperative CRP, and between ∆IL-6 and ∆CRP. The correlation analyses between IL-6 or CRP levels and anatomic features of cardiac myxomas proved that IL-6 levels were more sensitive than CRP in predicting the inflammatory status of cardiac myxoma patients.

Our study was confined to a limited number of patients and the results might have some biases owing to the lack of the necessary patient information. As no recurrent cardiac myxoma case was included in the present patient cohort, the predictive value of IL-6 in tumor recurrence was not evaluated.

## CONCLUSION

Anatomic features of cardiac myxomas (sessile, irregular appearance, maximal dimensions, and volumes) could be determinants of the patients' circulating IL-6 levels. In comparison to CRP, IL-6 was likely to be a more sensitive biomarker in predicting the inflammatory status of cardiac myxoma patients. Sessile and irregular cardiac myxomas might predict more severe inflammatory conditions for their more abundant endothelial cells owing to the enlarged dimension and tumor volume that are responsible for the overproduction of cytokines.

**Table:** 

Authors' roles & responsibilities	
SMY	Conception or design of the work; acquisition, analysis, or interpretation of data for the work; drafting the work or revising it critically for important intellectual content; final approval of the version to be published
HZL	Conception or design of the work; acquisition, analysis, or interpretation of data for the work; final approval of the version to be published
